# State Agencies’ Perspectives on Planning and Preparing for WIC Online Ordering Implementation

**DOI:** 10.3390/nu14214447

**Published:** 2022-10-22

**Authors:** Allison M. Nitto, Eric E. Calloway, Elizabeth T. Anderson Steeves, Amy Wieczorek Basl, Francesca Papa, Sarah K. Kersten, Jennie L. Hill

**Affiliations:** 1 Gretchen Swanson Center for Nutrition, Omaha, NE 68154, USA; 2 Population Health Sciences, University of Utah, Salt Lake City, UT 84108, USA

**Keywords:** WIC, online ordering, project implementation, nutrition assistance programs

## Abstract

Online ordering for the Special Supplemental Nutrition Program for Women, Infants, and Children (WIC) has the potential to alleviate some of the barriers faced by WIC participants when shopping with their WIC food benefits. WIC State agencies are the leaders in planning, preparing, implementing, maintaining, and expanding WIC online ordering. Cross-sectional web-based survey research was utilized to identify barriers to implementing WIC online ordering, as well as the support needed to overcome those barriers, from a WIC State agency perspective. Web surveys were administered to 81 WIC State agencies from 31 January 2022 to 1 April 2022. Descriptive statistics, independent samples *t*-test, and one-way analysis of variance were used to analyze the findings. Open-ended responses were analyzed using a qualitative iterative approach. WIC State agencies noted several barriers to implementing WIC online ordering, including limited staff capacity, WIC retailer interest, and technological capabilities.

## 1. Introduction

The Special Supplemental Nutrition Program for Women, Infants, and Children (WIC) is a critical public health program that served approximately 6.2 million low-income pregnant, breastfeeding, and non-breastfeeding post-partum women, as well as infants and children up to the age of 5, in 2021 [1,2]. WIC is administered by the U.S. Department of Agriculture’s Food and Nutrition Service (USDA-FNS) at the Federal level and through 89 WIC agencies at the State level, comprising the 50 States and the District of Columbia, 33 Indian Tribal Organizations, and 5 territories [2]. WIC benefits include supplemental food packages, nutrition education, breastfeeding support, and referrals to immunizations and other health services, among others [2]. The WIC food package supplements the diets of WIC participants with specific nutrients for a healthy and balanced diet [3]. Prior research has shown that participation in WIC leads to several positive outcomes, including birth outcomes, feeding and diet practices, cognitive development, immunization rates, and healthcare cost savings [4]. However, recent data has indicated that approximately 40% of the eligible WIC population does not participate in WIC [5], equating to millions of vulnerable women and children losing the key health benefits of WIC. Thus, enhancements to WIC that improve WIC participant enrollment and retention are critical, with recent research and funding initiatives focusing on enhancements to the WIC participant experience involving shopping and redeeming WIC food benefits [6].

Typically, WIC participants shop and redeem their WIC food benefits physically at WIC food retailers (i.e., WIC-authorized vendors), using their WIC electronic benefit transfer (EBT) card [7]. However, the in-store shopping experience has been linked to issues related to transportation, locating specific WIC-eligible foods in the store, and stigma concerns during the check-out process [8,9,10,11]. These issues with in-store shopping further intensified during the COVID-19 pandemic. Ordering and purchasing WIC foods online could help to alleviate these problems. In addition, a recent study has noted an increase in overall demand for online ordering and grocery delivery due to the COVID-19 pandemic [12].

While prior research on WIC online ordering is limited and small-scale, the available research has shown that online shopping may decrease in-store shopping time, and that online grocery platforms could make the identification and selection of WIC-approved items easier [13]. In addition, studies have shown that online ordering may improve perceived access to healthy foods, and may increase fruit and vegetable purchases among participants of Federal nutrition assistance programs [14]. Federal nutrition programs—including WIC—have started to progress towards online ordering of food benefits prior to and during the COVID-19 pandemic. The potential limitations related to WIC online ordering include the Federal requirements that WIC food benefits must be redeemed in the presence of a cashier and limitations on refunds and exchanges [15]; however, under the American Rescue Plan Act (PL 117-2; ARPA), a U.S. federal response to the COVID-19 pandemic, USDA-FNS is considering flexibilities submitted by WIC State agencies for WIC online ordering and transaction projects intended to promote outreach, innovation, and program modernization efforts [16].

WIC State agencies are leaders in the planning, preparing, implementing, maintaining, and expanding of WIC online ordering [6]. They also must coordinate with several partners, including their EBT processor (the technology contractor that manages WIC EBT payments) and WIC retailers, when pursuing WIC online ordering. Prior research on WIC stakeholders, including WIC State agencies, collected information regarding challenges with WIC online ordering from December 2020 to January 2021 [17]. However, since then, the USDA-FNS has released the ARPA flexibilities guidance and, so, we anticipated that some WIC State agencies could have further progressed with implementing WIC online ordering, resulting in lessons learned that could be useful to other WIC State agencies interested in WIC online ordering. Furthermore, additional time responding to the impacts of COVID-19 could have led WIC State agencies to identify other useful information related to WIC online ordering. In addition, in December 2021, the Gretchen Swanson Center for Nutrition (GSCN), in partnership with the USDA-FNS, has awarded competitive sub-grants to eight WIC State agencies, representing four unique sub-grant projects with multiple State agencies collaborating on one project, where the purpose of the sub-grants was to implement WIC online ordering projects. While the GSCN intends to collect evaluation data from the eight sub-grantees, information from the remaining 81 WIC State agencies on why they did not pursue support to implement WIC online ordering is unavailable. Some of these 81 WIC State agencies may also be in the process of planning, preparing, or implementing WIC online ordering. Thus, the purpose of this study was to address the following research questions: What barriers do WIC State agencies face when implementing WIC online ordering, what resources and support do WIC State agencies need to overcome those barriers, and what are the key lessons learned from State agencies that have experience with WIC online ordering? This information is crucial for the future expansion of WIC online ordering, ultimately improving WIC participant satisfaction and increasing participant access, redemption, and consumption of nutritious foods [13,14]. The following sections describe the materials and methods, results, discussion, and conclusion based on the defined study purpose, respectively.

## 2. Materials and Methods

### 2.1. Respondents

WIC operates through a total of 89 WIC State agencies, including 50 State health departments, the District of Columbia, 33 Indian Tribal Organizations (ITOs), and 5 U.S. territories (Northern Mariana, American Samoa, Guam, Puerto Rico, and the Virgin Islands). The eight WIC State agencies included in the GSCN WIC Online Ordering Sub-grant Project were not recruited to participate in the web-based survey. The survey was sent to a census of the remaining 81 WIC State agencies, representing all FNS regions, and included some WIC State agencies that were in some stage of implementing a WIC online ordering project. The web-based survey was sent to the WIC State Directors of the 81 State agencies; however, at the discretion of the WIC State Director, the survey link could be forwarded and completed by another knowledgeable staff member, such as the WIC Vendor Manager. WIC State agency director contact information was gathered from the USDA-FNS website.

### 2.2. Survey and Measures

A web-based survey was administered to capture insights on the feasibility and readiness of scaling out WIC online ordering to the 81 WIC State agencies beyond those eight included in the sub-grants. The survey instrument was designed using questions adapted from a validated survey [18]. The survey underwent thorough quality control checks before being finalized, in order to ensure that the survey was programmed accurately. The survey instrument, administered using the Qualtrics online survey software [19], included a total of 16 questions; however, due to programmed display logic and skip patterns, the respondents only received a subset of the questions. The survey instrument included both quantitative and qualitative (i.e., open text field) questions related to successes in and barriers to planning and implementing a WIC online ordering project (Table 1). The final survey instrument was estimated to take an average of 10–15 min. Close-ended survey questions asked about barriers impacting the implementation of WIC online ordering, reasons for not applying to the above-mentioned funding opportunity to support WIC online ordering implementation, and current progress made (if any) towards implementing WIC online ordering. Open-ended survey questions asked about steps needed to plan and implement a WIC online ordering system, key barriers and lessons learned through the planning and implementation of a WIC online ordering system, and conditions that are necessary for an existing WIC online ordering system to continue and expand.

### 2.3. Data Collection

The study was determined, by the University of Nebraska Medical Center Office of Regulatory Affairs, to be exempt from human subjects review (Exemption #0013-22-EX). Potential respondents were provided with information about the study on the first page of the survey, and electronic consent was obtained by respondents agreeing to proceed. Survey respondents were eligible to receive an incentive for completing the web-based survey, and had the option to choose to redeem a $20 gift card or donate $20 to a charitable organization. An email invitation to an electronic form of the survey was sent to 81 WIC State agencies on 31 January 2022. Data collection closed on 1 April 2022. Within this period, up to seven email and one telephone reminders were made to non-respondents. Data processing included data cleaning and data imputation or case-level edits, as needed, to prepare for analysis.

### 2.4. Analyses

#### 2.4.1. Assessing Risk Factors for Facing WIC Online Ordering Barriers

Respondents that reported not having a WIC Online Ordering project in progress were asked about 11 potential barriers related to WIC online ordering that they may have encountered, related to not having cooperation from various necessary partners, lack of interest, prohibitive costs, limited capacity, technology hurdles, and administrative and regulatory issues. For each of the barriers, respondents were asked if it was “not a factor”, “minor factor”, or “major factor” that was “… contributing to your State agency’s decision not to implement a WIC online ordering project”.

A reported barriers index score was created to quantify the barriers. Response options were scored 0 (not a factor), 1 (minor factor), or 2 (major factor), and summed for respondents with complete data. Therefore, a higher score indicates facing a larger number of reported barriers and/or facing more severe barriers. The internal consistency of the scale was above acceptable levels (Cronbach’s alpha = 0.82) [20].

WIC State agencies were categorized based on characteristics that may be relevant to implementing WIC online ordering. These categories were largely determined based on recent studies of perceived WIC online ordering challenges [17,21]. In those studies, factors such as rurality, internet access, and WIC retailer buy-in were cited factors that may be relevant to the implementation of WIC online ordering. Therefore, WIC State agencies in this study were categorized by State agency type (State, ITO, or territory); State agency size (small, <10,000 WIC participants; medium, 10,000–75,000; or large, >75,000; thresholds matched those used in the NSWP-III study [22]); being located within a State that is above the national median (25.6%) versus below the national median for percent of population living in rural areas [23]; being located within a State that is above the national median (63.6%) versus below the national median for internet access among low-income households (<$20,000 annual income) [24]; and being located within a State that is above the national median (0.137) versus below the national median for the number of WIC-authorized retailers per 1000 population (proxy measure for general retailer buy-in with the WIC program) [25]. Additionally, to assess potential regional differences, WIC State agencies were categorized by their FNS Regional Office coverage area: Mid-Atlantic, Midwest, Mountain Plains, Northeast, Southeast, Southwest, or Western.

Differences between barriers index scores across WIC State agency categories were assessed using an independent samples *t*-test (when two groups) and one-way analysis of variance (when three or more groups). An alpha level of 0.05 was used. Additionally, to further characterize between-group differences, effect sizes were calculated (Cohen’s d) with small (0.2), medium (0.5), and large (0.8) effect sizes described [26]. Additionally, the percentage of WIC State agencies citing each barrier (each of the 11 barriers included in the barriers index score) as a “major factor” was determined, both for the whole sample and by WIC State agency characteristic. The percentage of the whole sample that selected “not a factor”, “minor factor”, or “major factor” for each of the 11 barriers was also calculated.

#### 2.4.2. Reasons for Not Responding to the Funding Opportunity

The WIC State agencies in this study were asked if they considered applying to the above-mentioned funding opportunity, and why they ultimately did not apply for the funding (if applicable). Respondents were asked about 12 potential reasons for not applying, related to having enough information about the request for applications, having capacity to plan and prepare an application, being able to secure commitments from necessary partners, and being offered an appropriate funding level. Response options were “not a factor”, “minor factor”, or “major factor”. The analysis included calculating the proportion of respondents selecting “major factor” for not applying for all 12 reasons for the whole sample, stratified by those who considered applying (but did not) and those that did not consider applying.

#### 2.4.3. Qualitative Data Analysis

Qualitative data were collected from open-ended survey questions among those who responded to the survey. Additionally, two WIC State agencies who stated they did not have time to complete surveys provided responses regarding barriers to WIC online ordering over the phone or email, which were included in the qualitative analysis of open-ended questions regarding barriers. Qualitative data were analyzed using Microsoft Excel [27]. The researchers used an iterative approach [28] to develop an analytic codebook. Verbatim responses were first coded for categories, patterns, and themes. Further refinement of the analytic codebook occurred during coding. Three researchers trained in qualitative analysis coded open-ended text responses individually, then met to discuss their findings and reconcile differences until the inter-rater reliability agreement reached >95%. Quotations were selected to illustrate and provide depth to the findings.

## 3. Results

### 3.1. WIC State Agency Characteristics

A total of 58 WIC State agencies out of the 81 invited responded to all survey questions (72% overall response rate: 33/44 invited States/DC [75%]; 22/32 invited ITOs [69%]; 3/5 invited territories [60%]). A total of 47 respondents provided data to open-ended survey items and were included in the qualitative sample. All seven FNS Regional Offices were represented in the sample. Respondents mostly represented States, rather than ITOs or territories, and most had caseloads greater than 10,000 WIC participants. Additional sample characteristics are described in Table 2. Two WIC State agencies reported having already begun implementing WIC online ordering of some form in their State. Additionally, six WIC State agencies reported that they were planning to implement WIC online ordering within the next 6–24 months.

### 3.2. Barriers to Implementing WIC Online Ordering

The 56 WIC State agencies that reported not implementing WIC online ordering were asked about the barriers faced. On average, respondents scored 4.8 (SD = 4.3) on the barriers scale, with a range from 0.0–18.0 (note: a higher score indicates that a WIC State agency experiences more barriers and/or more impactful barriers). Table 3 shows the scores for the barriers scale by WIC State agency characteristics. There were no statistically significant findings, likely due to the small sample size. However, there were several “small” to “large” effect sizes. WIC State agencies in ITOs or territories, within the Mountain Plains FNS Regional Office, with a caseload below 10,000, within a more rural State, and/or with more WIC retailers per 1000 population saw at least “small” effect sizes for increased barriers scale scores.

**Figure 1:**
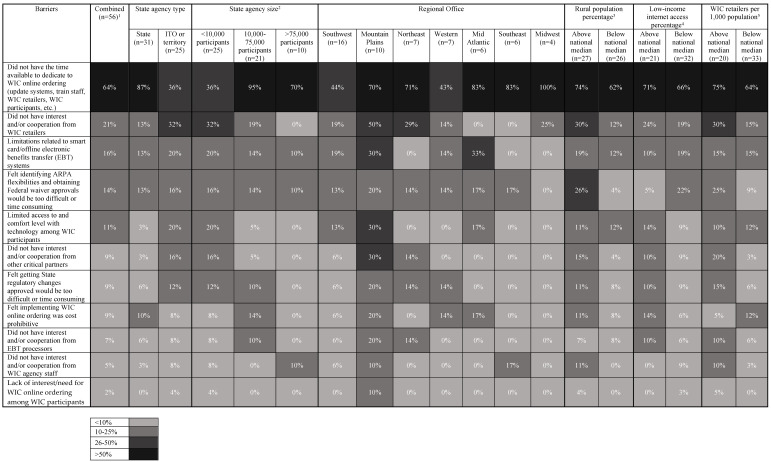
Percentage of Respondents Reporting Barriers being a “Major Factor” Preventing Implementation of WIC Online Ordering by WIC State Agency Characteristics (n = 56). WIC: Special Supplemental Nutrition Program for Women, Infants, and Children; ARPA: American Rescue Plan Act; EBT: electronic benefit transfer. ^1^ 56 State agencies responded to the survey and had not implemented a WIC Online Ordering project. Sample size is 53, rather than 56, when there is no secondary data for the three territories. ^2^ 2021 WIC State Agency Participation Data [29]. ^3^ 2010 Census Data [23]. ^4^ 2019 American Community Survey Data [24]. ^5^ 2016 Food Environment Atlas Data [25].

Figure 1 shows the specific barriers and the percentage of respondents reporting the barriers as a major factor impeding their implementation of WIC online ordering (Table A1 additionally shows the breakdown between “major”, “minor”, and “not a factor” for each barrier for the full sample). Overall, 64% of WIC State agencies endorsed “Did not have the time available to dedicate to WIC online ordering (update systems, train staff, vendors [retailers], WIC participants, etc.)” as a major factor; this was the most frequently cited barrier. Other frequently cited barriers were “Did not have interest and/or cooperation from vendors [retailers]” (21%), “Limitations related to smart card/offline electronic benefits transfer (EBT) systems” (16%), and “Felt obtaining Federal waiver approvals would be too difficult or time consuming” (14%). The three least frequently endorsed barriers were “Did not have interest and/or cooperation from EBT processors” (7%), “Did not have interest and/or cooperation from agency staff” (5%), and “Lack of interest/need for WIC online ordering among WIC participants” (2%).

As part of open-ended questions, some WIC State agencies provided additional insights on the barriers to implementing WIC online ordering. Competing priorities also influenced the time the WIC State agency had to implement WIC online ordering. Specific mentions of competing priorities included having currently or recently implemented an EBT system (n = 5) and currently or recently having made changes to their Management Information System (MIS; n = 6), with a WIC State agency explaining that they “did not have the capacity to implement and/or manage a WIC online ordering project while … in the process of transferring and implementing a new Management Information System”. For barriers related to WIC retailers, four WIC State agencies indicated that they met with or had tried to meet with large chain retailers; however, they were not interested, due to the risk in investing in a system that they felt may not be supported or meet Federal regulations in the long-term. For instance, a WIC State agency stated “[it’s] hard to get a grocery store to commit to do something [temporarily]. Grocery stores felt it was too much of a risk”. As for technology updates, State agencies reported needing changes to one or more of the following systems: EBT system (n = 14), MIS system (n = 9), and WIC retailer online ordering platform (n = 2).

When looking across WIC State agency categories in Figure 1, interesting patterns emerged. Fewer ITOs/territories (36%), compared to States (87%), indicated not having time available to dedicate to WIC online ordering as a major barrier. For ITOs/territories, not having interest from WIC retailers (32%) and working with WIC participants who were less familiar with/had lower access to technology needed for online ordering (20%) were more frequently reported barriers than for States (13% and 3%, respectively). ITOs/territories made up all the <10,000 caseload category, so findings by WIC State agency size largely mirrored the ITO/territory versus State findings. The Southwest and Mountain Plains FNS Regional Offices largely comprised ITOs in this sample; however, they showed different patterns of barriers. Mountain Plains more frequently reported barriers overall, compared to the Southwest, and frequently reported not having time available to dedicate to WIC online ordering (70% versus 44%, respectively), not having interest from WIC retailers (50% versus 19%, respectively), and not having interest from other critical partners (30% versus 6%, respectively) as major barriers. WIC State agencies in more rural States, compared to less rural States, more frequently reported not having interest from WIC retailers (30% versus 12%, respectively), and felt that identifying ARPA flexibilities and obtaining Federal waiver approvals would be too difficult or time-consuming (26% versus 4%, respectively). WIC State agencies in States below the national median for low-income internet access, compared to those above the median, more frequently reported that identifying ARPA flexibilities and obtaining Federal waiver approvals would be too difficult or time-consuming (22% versus 5%, respectively). Finally, WIC State agencies with more per capita WIC retailers, compared to those with fewer, more frequently reported not having interest from WIC retailers (30% versus 15%, respectively), that identifying ARPA flexibilities and obtaining Federal waiver approvals would be too difficult or time-consuming (25% versus 9%, respectively), and did not have interest from other critical partners (20% versus 3%, respectively). Many WIC State agencies (14 out of 20) above the median for per capita WIC retailers were also in States above the median for rural population percentage, so the patterns of barriers were similar when comparing WIC State agencies by rurality and WIC retailers per capita.

### 3.3. Reasons for Not Applying to WIC Online Ordering Funding Opportunity

Among the full survey sample (n = 58), the top three reasons for not applying to the GSCN WIC Online Ordering Sub-grant Project funding opportunity were concerns around capacity, including limited capacity to implement and manage the project (71%), to develop a proposal (55%), and to prepare implementation plans (47%); see Table A2. The least-frequently reported reasons for not applying were not having enough information (10%), too little funding provided (9%), and did not need funding (5%). The group that considered applying, but did not (n = 25), and the group that did not consider applying (n = 33) did not vary greatly in their patterns of responses; other than the ‘did not consider applying’ group presenting a higher frequency (39%) of reporting not having the technology to implement WIC online ordering (e.g., WIC State agencies using smart cards or have not implemented WIC EBT), compared to the group that considered applying (20%).

### 3.4. Best Practices and Strategies for WIC Online Ordering

The survey also included open-ended questions on best practices and successful strategies for planning, preparing, and implementing WIC online ordering. Please note that these questions were answered by a smaller subset of the WIC State agencies that had reported some sort of experience with WIC online ordering. A total of 15 respondents shared at least one step that their WIC State agency has taken or will need to take to prepare for WIC online ordering. The most common preparation and planning step was working with partners. The range of partners listed included WIC retailers, EBT processors, other WIC State agencies, USDA Regional Offices, internal IT departments, the National FNS Office, and other types of partners, such as food desert delivery retailers, WIC local agencies, and WIC clinics. One WIC State agency expressed that “… staying involved, starting with vendor[retailer]-participant relations all the way down the line to Local Agency, [retailer], and State Office teamwork and communication has been essential”. The next most-reported steps included developing a plan, understanding the technology in place/needed/challenges, navigating approval processes, and training groups such as WIC staff, WIC retailers, or WIC participants; for example, one WIC State agency stated that “clinics need to be prepared to provide thorough training to participants to successfully use the ordering system”.

Three respondents with experience in planning, preparing, and/or implementing WIC online ordering projects shared successful strategies used when starting and/or implementing an online ordering system. The most common successful strategy was clear and frequent communication between partners with a WIC State agency, indicating “constant communication is important with vendors [retailers], EBT processor, Region Management, [and] local agency”. A total of six respondents indicated several conditions (i.e., inputs) needed to maintain and/or expand an online ordering system. The most common conditions cited were buy-in from the WIC retailer, WIC participant training and technical assistance, and adequate technology systems. One WIC State agency indicated that “a reliable and committed retail partner [is an important condition for online ordering]” and another State agency stated that “having better technology that works with vendors’ [retailers’] systems” is important. Additional themes emerged, including the need for financial support and FNS support (through policy change).

## 4. Discussion

We identified several barriers to implementing WIC online ordering projects, with lack of time available to dedicate to WIC online ordering as a major factor reported by most WIC State agencies. A similar response was reported when WIC State agencies were asked about reasons that they did not apply for the GSCN WIC Online Ordering Sub-grant Project with WIC State agencies, indicating that they did not have the capacity to draft the proposal for the grant opportunity. USDA-FNS often releases grant funding mechanisms to help WIC State and local agencies to enhance their program operations [30]. Unfortunately, the same WIC agencies might be missing out on these opportunities if limited staffing is a recurring issue. Funding mechanisms could be a potential way to help support WIC State agencies in hiring the necessary staff to dedicate to WIC online ordering implementation (assuming the WIC State agency can allocate the time for application process); however, as with most grant funding mechanisms, the funds are set with a fixed price and expiration date. Thus, the WIC State agency’s budget outside of the grant funding will need to account for the ongoing maintenance cost of WIC online ordering. The GSCN WIC Online Ordering Sub-grant Project intends to collect information on staffing needs and project costs, in order to estimate potential future maintenance costs. This information will be shared with WIC State agencies, beyond just those participating in the sub-grants, as a tool to help them plan and budget for WIC online ordering.

Another major barrier to WIC online ordering implementation focused on a lack of interest and commitment from WIC retailers. WIC retailers are an integral partner in the WIC online ordering process, and their support and engagement are necessary for implementation. Organizations such as the National WIC Association, National Grocers Association (NGA), and so on, could help WIC State agencies in increasing retailer awareness and support for WIC online ordering. While the average monthly WIC food benefit dollar amount is an estimated 16% of the average monthly SNAP (Supplemental Nutrition Assistance Program) food dollar amount [31], approximately 50% of WIC participants participate in SNAP [32]. In addition, nearly 20% of children receiving SNAP benefits also receive WIC benefits [33]. Therefore, offering both SNAP and WIC online ordering could help increase WIC and SNAP customer satisfaction, as well as their likelihood to use both SNAP and WIC benefits online at the same retailer. In addition, WIC retailers could be waiting to financially invest in WIC online ordering, depending on whether the current ARPA flexibilities become permanent after the public health emergency ends. Thus, Federal action on extending the ARPA flexibilities, such as removing the requirement for cashier presence for WIC transactions, is important for the future of WIC online ordering

Another key implementation challenge focused on WIC State agencies that used smart cards for transactions of WIC food benefits, as offline smart card systems cannot facilitate WIC online ordering with online transactions [7]. At present, 16 of the 89 WIC State agencies use offline EBT systems [34]. On the other hand, WIC State agencies with online systems use an EBT with a magnetic strip connected to an online system that has benefit information in real-time, enabling WIC online ordering with online transactions. The current level of interest or plans to update to an online system is unknown among the 16 WIC offline EBT State agencies. However, if they do change to an online system, that change will likely require significant time and staffing resources. As technology continues to advance and evolve, it is likely that future enhancements to WIC systems, including and beyond those related to WIC online ordering, would most likely be based on an online system. Thus, WIC State agencies with offline EBT systems might consider the transition to online systems as part of their future strategic plans related to technological efforts. In addition, external funding opportunities, such as Federal grants, could help to support WIC State agencies in transitioning to online systems.

Importantly, a lack of interest or need for WIC online ordering among WIC participants was reported by only 2% of WIC State agencies, suggesting that survey respondents felt that WIC participants were overall interested and/or wished to shop for their WIC food benefits online. This finding aligns with the literature showing that WIC participants are interested in technological solutions, including online ordering with WIC [13,35,36]. This also stresses the role of WIC online ordering in increasing WIC participant enrollment, satisfaction, and retention in the WIC program. Recent data has shown that only 57% of eligible WIC participants are enrolled in WIC [5], and the in-store WIC shopping experience has been reported as a reason for WIC participants leaving WIC [37]. Hence, program enhancements—such as providing WIC participants with the option to shop for their food benefits online—are important factors in the overall success and reach of WIC.

At present, the GSCN WIC online ordering implementation guide, titled the “Blueprint for WIC Online Ordering Projects” (and known as the “Blueprint”) and released in June 2021, is a key resource for WIC State agencies planning to implement WIC online ordering [6]. The Blueprint is based on WIC expert feedback and consensus building. At the conclusion of the GSCN WIC Online Ordering Sub-grant Project, the Blueprint will be updated based on the robust evaluation conducted by GSCN. This evaluation will explore key aspects of WIC online ordering and transactions, such as system-level factors related to successful adoption and implementation, costs, and overall impact, using the RE-AIM (Reach, Effectiveness, Adoption, Implementation, and Maintenance) framework—a program planning and evaluation framework that can be used to determine the effectiveness of an initiative which considers factors associated with external validity, such as adoption and implementation [38].

Future research should consider the ability of local WIC agencies to implement WIC online ordering, including their capacity to communicate changes to WIC participants in collaboration with local retailers who have adopted WIC online ordering. Future research could also investigate barriers to WIC online ordering adoption and implementation among those WIC retailers not participating in the GSCN WIC Online Ordering Sub-grant Project. Based on the feedback from the WIC State agencies, commitment from WIC retailers was a key barrier in WIC State agencies moving forward with WIC online ordering implementation. Thus, research could be beneficial to helping identify and resolve any unknown challenges among WIC retailers in implementing WIC online ordering. Another key gap in WIC online ordering research to date is research including WIC participants. There have been small-scale studies in WIC participants on their interest and utilization of WIC online ordering [13,36,39], and the GSCN WIC Online Ordering Sub-grant Project intends to incorporate feedback from WIC participants; however, data from a large, diverse sample of WIC participants on WIC online ordering is currently unavailable. Incorporating the end-user perspective in each stage of the WIC online ordering process is critical to enhancing the future reach, effectiveness, and sustainability of WIC online ordering. Finally, additional research on the equity of WIC online ordering availability at the WIC retailer and participant level also is needed to help avoid creating inequitable access of online ordering for WIC participants. Examining such inequities could ensure that online ordering platforms are available in multiple languages for all WIC populations to access, or that geographic disparities are not created based on select retailers making WIC online ordering available. Future research on the equity of WIC online ordering could help to reduce or eliminate unintended consequences, ensuring that WIC online ordering is accessible for all WIC participants.

This study had a couple limitations to note. First, the survey only captured the perspective of WIC State agencies and did not include feedback from other key stakeholders involved in WIC online ordering, such as WIC retailers, WIC participants, WIC local agencies, EBT processors, and so on. In addition, we were unable to collect detailed information on key lessons learned and solutions to overcoming challenges when implementing WIC online ordering, despite including questions focused on those areas, as the open-ended questions were only asked to the eight WIC State agencies with existing or planned WIC online ordering projects. These WIC State agencies provided limited information in response to those questions, likely due to their limited experience with WIC online ordering thus far. WIC State agencies might have more detailed information to provide in the future, as they progress with implementing WIC online ordering. The findings from this study were also supported by several strengths, including successfully recruiting WIC State agencies with a 72% response rate. This allowed for the reporting of findings from a diverse group of WIC State agencies. Furthermore, the large number of responses allowed for additional analysis by sub-groups, providing important insights regarding the barriers to implementing WIC online ordering with respect to the characteristics of WIC State agencies.

## 5. Conclusions

The findings presented in this study highlight the key challenges to implementing WIC online ordering from a WIC State agency perspective. WIC State agencies noted several barriers to implementing WIC online ordering, including limited staff capacity, WIC retailer interest, and technological capabilities. Additional WIC State agency staff dedicated to WIC online ordering and funding to help modernize their technology systems would be beneficial, helping to overcome these challenges. Furthermore, WIC State agencies could use assistance from external partners, in terms of gaining WIC retailer support and commitment to WIC online ordering. Future research on WIC online ordering projects, including and beyond the GSCN WIC Online Ordering Sub-grant Project, is crucial for the future success of WIC online ordering. Potential areas of future research include assessing the key successes and challenges related to implementing WIC online ordering from both the perspective of local agencies and retailers, evaluating the end-user experience with WIC online ordering in a large and diverse sample of WIC participants, and investigating equitable access to WIC online ordering. As WIC online ordering is a relatively new endeavor in a limited number of State agencies, continued data collection from WIC agencies, retailers, participants, and other stakeholders is important for the future success and reach of WIC online ordering.

## Figures and Tables

**Table 1:** Summary of Survey Questions.

Introduction Questions
Did your WIC State agency consider applying for the WIC Online Ordering Sub-grant funding opportunity?
What steps, if any, did your State agency take to assess interest and/or feasibility for pursuing the WIC Online Ordering Sub-grant Project funding opportunity? Select all that apply.
Is your State agency already implementing or planning to implement a WIC online ordering project?
What is your State agency’s anticipated timeline for starting a WIC online ordering project?
Please indicate whether each of the following was not a factor, a minor factor, or a major factor contributing to your State agency’s decision NOT to apply for the WIC Online Ordering Sub-grant Project funding. Response options include N/A, Not a Factor, Minor Factor, Major Factor.Examples of factors assessed: Did not have enough information about the application process Did not find requirements of participation in the sub-grant project appealing (too burdensome, not interesting, etc.) Did not think that enough funding was available (i.e., award dollar amount was too low) Did not have the capacity to implement and/or manage a WIC online ordering project
We are also interested in reasons for not implementing a WIC online ordering project outside of the GSCN sub-grant opportunity. Please indicate whether each of the following was not a factor, a minor factor, or a major factor contributing to your State agency’s decision NOT to implement a WIC online ordering project. Response options include N/A, Not a Factor, Minor Factor, Major Factor.Examples of factors assessed: Did not have interest and/or cooperation from agency staff Did not have interest and/or cooperation from EBT processors Did not have interest and/or cooperation from other critical partners Felt obtaining Federal waiver approvals would be too difficult or time consuming
Existing WIC Ordering Projects Block: Questions were only asked for those who responded ‘yes’ to already implementing or was planning to implement a WIC online ordering project.
When did your State agency start planning and implementing a WIC online ordering project?
Select all organizations that your State agency engaged with to plan and implement your WIC online ordering project.Examples of organizations included: USDA FNS WIC local agency or clinic Other WIC State agency(ies) WIC participants
What steps did your State agency take to plan and prepare for your WIC online ordering project?
Please describe the top three challenges or barriers your State agency has faced in planning and implementing your WIC online ordering project and how you overcome those challenges.
Based on your experiences with your current WIC online ordering project, what key lessons learned would you like to share with other State agencies that are considering implementing their own project (e.g., important project components, beneficial implementation practices, key partnerships, etc.).
What conditions are necessary for your WIC online ordering project to continue successfully?
Thinking about the future of your WIC online ordering project, what conditions are necessary for the expansion?

**Table 2:** Responding WIC State Agency Characteristics (n = 58) ^1^.

Variable	Category	Frequency
State agency type	State	33
	ITO	22
	Territory	3
State agency size ^2^	<10,000 participants	25
	10,000–75,000 participants	23
	>75,000 participants	10
Regional Office	Southwest	17
	Mountain Plains	10
	Western	8
	Northeast	7
	Mid Atlantic	6
	Southeast	6
	Midwest	4
Rural population percentage ^3^	Above national median	28
	Below national median	27
Low-income internet access percentage ^4^	Above national median	21
	Below national median	34
WIC retailers per 1000 population ^5^	Above national median	21
	Below national median	34

^1^ 58 WIC State agencies provided complete survey data. The sample size is 55 in some cases, where secondary data could not be obtained for three territories. ^2^ 2021 WIC State Agency Participation Data [29]. ^3^ 2010 Census Data [23]. ^4^ 2019 American Community Survey Data [24]. ^5^ 2016 Food Environment Atlas Data [25].

**Table 3:** Barriers Scores by WIC State Agency Characteristics (n = 56) ^1^.

Agency Characteristic		Mean Score	SD	N	*p*-Value ^2^	Cohen’s d
State agency type	State	4.26	2.77	31	0.367	0.254
	ITO or territory	5.40	5.72	25		
State agency size ^3,4,5^	<10,000 participants	5.40	5.72	25	0.372	0.528
	10,000–75,000 participants	4.81	2.86	21		
	>75,000 participants	3.10	2.28	10		
Regional Office ^4,6^	Southwest	3.94	4.68	16	0.151	0.938
	Mountain Plains	8.50	5.89	10		
	Northeast	4.29	3.55	7		
	Western	3.43	3.26	7		
	Mid Atlantic	4.50	2.43	6		
	Southeast	3.67	2.34	6		
	Midwest	4.00	2.71	4		
Rural population percentage ^7^	Above national median	5.78	5.21	27	0.157	0.393
	Below national median	4.08	3.20	26		
Low-income internet access percentage ^8^	Above national median	5.10	3.18	21	0.825	0.061
	Below national median	4.84	5.07	32		
WIC retailers per 1000 population ^9^	Above national median	6.30	3.94	20	0.142	0.565
	Below national median	4.12	3.77	33		

^1^ 56 State agencies responded to the survey and had not implemented a WIC Online Ordering project. Sample size is 53, rather than 56, when there is no secondary data for the three territories. ^2^ Independent samples t-test to assess group differences, unless otherwise indicated. ^3^ 2021 WIC State Agency Participation Data [29]. ^4^ One-way analysis of variance used to assess group differences. ^5^ Effect size comparing <10,000 to >75,000. ^6^ Effect size for being located in the Mountain Plains region versus not. ^7^ 2010 Census Data [23]. ^8^ 2019 American Community Survey Data [24]. ^9^ 2016 Food Environment Atlas Data [25].

## Data Availability

Aggregate, deidentified data that support the findings of this study are available from the corresponding author, AN, upon written, reasonable request.

## Appendix A

**Table A1:** Reported Barriers to Implementing WIC Online Ordering in Order from Highest Percentage “Major Factor” (top) to Lowest Percentage “Major Factor” (bottom) (n = 56).

Importance	Frequency	Percentage
Did not have the time available to dedicate to WIC online ordering (update systems, train staff, WIC retailers, WIC participants, etc.)		
“Not a factor”	14	25
“Minor factor”	6	11
“Major factor”	36	64
Did not have interest and/or cooperation from WIC retailers		
“Not a factor”	42	75
“Minor factor”	2	4
“Major factor”	12	21
Limitations related to smart card/offline electronic benefits transfer (EBT) systems		
“Not a factor”	41	73
“Minor factor”	6	11
“Major factor”	9	16
Felt identifying ARPA flexibilities and obtaining Federal waiver approvals would be too difficult or time consuming		
“Not a factor”	33	59
“Minor factor”	15	27
“Major factor”	8	1
Limited access to and comfort level with technology among WIC participants		
“Not a factor”	42	75
“Minor factor”	8	14
“Major factor”	6	11
Did not have interest and/or cooperation from other critical partners		
“Not a factor”	46	8
“Minor factor”	5	9
“Major factor”	5	9
Felt getting State regulatory changes approved would be too difficult or time consuming		
“Not a factor”	41	73
“Minor factor”	10	18
“Major factor”	5	9
Felt implementing WIC online ordering was cost prohibitive		
“Not a factor”	38	68
“Minor factor”	13	23
“Major factor”	5	9
Did not have interest and/or cooperation from EBT processors		
“Not a factor”	48	86
“Minor factor”	4	7
“Major factor”	4	7
Did not have interest and/or cooperation from WIC agency staff		
“Not a factor”	49	88
“Minor factor”	4	7
“Major factor”	3	5
Lack of interest/need for WIC online ordering among WIC participants		
“Not a factor”	49	88
“Minor factor”	6	11
“Major factor”	1	2

**Table A2:** Percentage of Respondents that Selected “Major Factor” for Reasons for Not Applying to the GSCN WIC Online Ordering Sub-grant Project Funding Opportunity (n = 58).

Reason Selected	Considered Applying, But Did Not (n = 25)	Did Not Consider Applying (n = 33)	Combined (n = 58)
Did not have the capacity to implement and/or manage a WIC online ordering project	76%	67%	71%
Did not have staff capacity and/or expertise on staff to develop a proposal	60%	52%	55%
Did not have the capacity to prepare adequate implementation plans needed for the proposal	48%	45%	47%
Did not have enough time to prepare and submit a proposal (i.e., time between funding announcement and deadline was too short)	40%	33%	36%
Did not have the technology to implement a WIC online ordering project	20%	39%	31%
Did not receive commitment from a WIC retailer	24%	21%	22%
Felt identifying ARPA flexibilities and obtaining Federal waiver approvals would be too difficult or time consuming	28%	18%	22%
Did not find requirements of participation in the sub-grant project appealing (too burdensome, not interesting, etc.)	20%	18%	19%
Did not receive commitment from an EBT processor	16%	12%	14%
Did not have enough information about the application process	8%	12%	10%
Did not think that enough funding was available (i.e., award dollar amount was too low)	16%	3%	9%
Did not need the funding	4%	6%	5%
